# QuiCAT: a scalable and flexible framework for mapping synthetic sequences

**DOI:** 10.1093/bioinformatics/btaf607

**Published:** 2025-11-11

**Authors:** Daniele Lucarelli, Tina Kos, Caylie Shull, Sara Jiménez, Rupert Öllinger, Roland Rad, Dieter Saur, Fabian J Theis

**Affiliations:** Institute of Computational Biology, Helmholtz Center, Munich, Germany; Division of Translational Cancer Research, German Cancer Research Center (DKFZ), German Cancer Consortium (DKTK), Heidelberg, 69120, Germany; Chair of Translational Cancer Research and Institute of Experimental Cancer Therapy, Klinikum rechts der Isar, TUM School of Medicine and Health, Technical University of Munich, Munich, Germany; Center for Translational Cancer Research (TranslaTUM), TUM School of Medicine and Health, Technical University of Munich, Munich, Germany; German Cancer Consortium (DKTK), partner site Munich, a partnership between DKFZ and TUM University Hospital Klinikum rechts der Isar, Munich, Germany; Division of Translational Cancer Research, German Cancer Research Center (DKFZ), German Cancer Consortium (DKTK), Heidelberg, 69120, Germany; Chair of Translational Cancer Research and Institute of Experimental Cancer Therapy, Klinikum rechts der Isar, TUM School of Medicine and Health, Technical University of Munich, Munich, Germany; Center for Translational Cancer Research (TranslaTUM), TUM School of Medicine and Health, Technical University of Munich, Munich, Germany; German Cancer Consortium (DKTK), partner site Munich, a partnership between DKFZ and TUM University Hospital Klinikum rechts der Isar, Munich, Germany; Division of Translational Cancer Research, German Cancer Research Center (DKFZ), German Cancer Consortium (DKTK), Heidelberg, 69120, Germany; Chair of Translational Cancer Research and Institute of Experimental Cancer Therapy, Klinikum rechts der Isar, TUM School of Medicine and Health, Technical University of Munich, Munich, Germany; Center for Translational Cancer Research (TranslaTUM), TUM School of Medicine and Health, Technical University of Munich, Munich, Germany; Institute of Computational Biology, Helmholtz Center, Munich, Germany; Center for Translational Cancer Research (TranslaTUM), TUM School of Medicine and Health, Technical University of Munich, Munich, Germany; Department of Internal Medicine II, TUM School of Medicine and Health, Technical University of Munich, Munich, Germany; Center for Translational Cancer Research (TranslaTUM), TUM School of Medicine and Health, Technical University of Munich, Munich, Germany; German Cancer Consortium (DKTK), partner site Munich, a partnership between DKFZ and TUM University Hospital Klinikum rechts der Isar, Munich, Germany; Department of Internal Medicine II, TUM School of Medicine and Health, Technical University of Munich, Munich, Germany; Division of Translational Cancer Research, German Cancer Research Center (DKFZ), German Cancer Consortium (DKTK), Heidelberg, 69120, Germany; Chair of Translational Cancer Research and Institute of Experimental Cancer Therapy, Klinikum rechts der Isar, TUM School of Medicine and Health, Technical University of Munich, Munich, Germany; Center for Translational Cancer Research (TranslaTUM), TUM School of Medicine and Health, Technical University of Munich, Munich, Germany; German Cancer Consortium (DKTK), partner site Munich, a partnership between DKFZ and TUM University Hospital Klinikum rechts der Isar, Munich, Germany; Department of Internal Medicine II, TUM School of Medicine and Health, Technical University of Munich, Munich, Germany; Institute of Computational Biology, Helmholtz Center, Munich, Germany; School of Computing, Information and Technology, Technical University of Munich, Munich, Germany; TUM School of Life Sciences Weihenstephan, Technical University of Munich, Munich, Germany

## Abstract

**Motivation:**

Synthetic cellular tagging technologies play a crucial role in cell fate and lineage-tracing studies. Their integration with single-cell and spatial transcriptomics assays has heightened the need for scalable software solutions to analyze such data. However, previous methods are either designed for a subset of tagging technologies, or lack the performance needed for large-scale applications.

**Results:**

To address these challenges, we developed Quick Clonal Analysis Toolkit (QuiCAT), an end-to-end Python-based package that streamlines the extraction, clustering, and analysis of synthetic tags from sequencing data. QuiCAT outperforms existing pipelines in both speed and accuracy. Its outputs are widely compatible with the Python ecosystem for single-cell and spatial transcriptomics data analysis packages allowing seamless integrations and downstream analyses. QuiCAT provides users with two workflows: a reference-free approach for extracting and mapping synthetic tags, and a reference-based approach for aligning tags against known sequences. We validate QuiCAT across diverse datasets, including population-level data, single-cell and spatially resolved transcriptomics, and benchmarked it against the two most recently published tools. Our computational optimizations enhance performance while improving accuracy.

**Availability:**

QuiCAT is available as a Python package to be installed. The source code is available at https://github.com/theislab/quicat

## 1 Introduction

The need to differentiate and track individual cells or cell populations has driven the development of various cellular barcoding systems over the past years (Lu *et al*. 2011; Biddy *et al*. 2018; Wroblewska *et al*. 2018; Weinreb *et al*. 2020; Emert *et al*. 2021; Oren *et al*. 2021; Chang *et al*. 2022; Fennell *et al*. 2022; Ratz *et al*. 2022; Umeki *et al*. 2022; Yalcin *et al*. 2023; Baygin *et al*. 2024; Jang *et al*. 2025; Kinsler *et al*. 2025). These barcoding techniques allow researchers to quantify the relative abundance of uniquely barcoded cells in samples using DNA sequencing. When paired with phenotypic readouts like single-cell RNA sequencing (scRNA-seq) and spatially resolved transcriptomics (SRT), synthetic barcodes enable the tracking and characterization of distinct cell populations and their behaviours across various experimental conditions (Serrano *et al*. 2022). Recent advancements led to an increased affordability and accessibility of scRNA-seq, allowing more research groups to adopt barcoding systems for investigating heterogeneous cell populations (Serrano *et al*. 2022; Howland and Brock 2023). However, these systems are often developed independently and tailored for specific applications. This has resulted in an array of barcode structures and analysis software packages, which are typically optimized for particular designs (Corsello *et al*. 2020; Weinreb *et al*. 2020; Ratz *et al*. 2022; Umeki *et al*. 2022; Baygin *et al*. 2024; Jindal *et al*. 2024; Jang *et al*. 2025; Kinsler et al. 2025). For example, many available pipelines have limited flexibility in terms of barcode length and structure, restricting the range of potential applications. Several others are compatible with only a narrow subset of sequencing technologies, and exclusively accept FASTQ input files. In response to these challenges, two more flexible software packages have recently emerged to accommodate a range of barcoding techniques (Daylin and Amy 2024; Holze *et al*. 2024). Despite these advances, they often fall short in terms of sequence flexibility, supported technologies, and, importantly, overall performance. Most pipelines suffer from suboptimal processing speeds, making them unsuitable for massively parallel screens—key applications of synthetic tagging systems as the field shifts toward large-scale, high-throughput approaches (Zhang *et al*. 2025). Most of them function primarily as wrappers around pre-existing tools, lacking computational optimizations. Moreover, the majority of the other pipelines do not support reference-based barcode extraction methods. Collectively, these constraints create a fragmented landscape forcing users to navigate the complexity and select the tool that fits each unique application.

**Figure 1.**
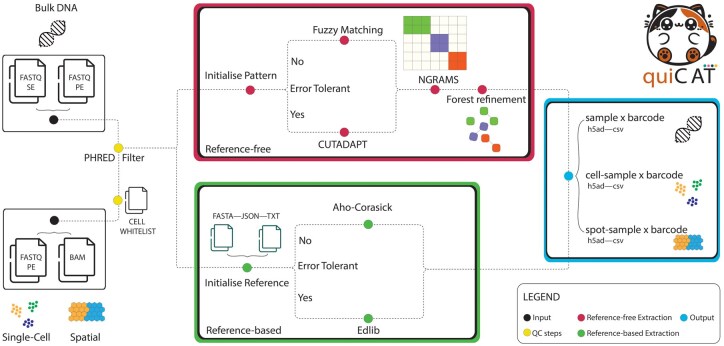
Overview of QuiCAT extraction workflow. Bulk DNA sequencing, scRNA-seq, or SRT FASTQ, or BAM files are ingested as inputs (black). Reads are filtered based on quality thresholds and whitelisted cells/spots (yellow). Barcodes are extracted using either a reference-based (green) or reference-free (red) workflow. The final AnnData and CSV outputs containing count matrices (blue) can be used for downstream analysis and visualization.

To address this gap, we introduce Quick Clonal Analysis Toolkit (QuiCAT), a Python-based toolkit designed for rapid, accurate, and flexible barcode extraction and analysis from sequencing data (Fig. 1). Regardless of barcode length or structure, QuiCAT’s flexibility allows it to adapt to any barcoding library used and to generate count matrices from population-based DNA, scRNA-seq, and SRT data. QuiCAT supports multiple sequencing technologies and accepts diverse input formats, offering both reference-free and reference-based extraction methods. We benchmarked QuiCAT against the current state-of-the-art pipelines using a publicly available population-based dataset, and a synthetically generated dataset. Additionally, we applied QuiCAT to capture combinatorial barcodes in a newly generated dataset featuring Pro-codes (Wroblewska *et al*. 2018; Dhainaut et al. 2022) combined with scRNA-seq, and a publicly available SRT dataset (Ratz *et al*. 2022).

## 2 Methods

### 2.1 Quicat overview

QuiCAT is a high-performance, flexible, and scalable Python-based package for the extraction of synthetic barcodes from sequencing data. Its modular design allows efficient retrieval from different modalities, including DNA sequencing, scRNAseq or SRT datasets. QuiCAT is built from the ground up to enhance efficiency, reduce redundant computations, and scale effectively for large datasets.

At the core of QuiCAT is the extraction workflow (Fig. 1 and Supplementary Fig. 1), which supports both binary alignment map (BAM) and FASTQ input files, accommodating paired-end or single-end reads. If starting from BAM files, the user has an option to scan all reads, to only scan reads aligned to specific contigs, or to scan all the unaligned reads where the synthetic barcodes are usually found leaving the user full flexibility. The workflow begins with a QC step that removes reads failing user-defined QC criteria. The remaining reads are then scanned for sequences of interest using either a reference-based or a reference-free approach. In reference-based mode, users supply a list of known barcode sequences for exact matching, allowing precise retrieval. Typically, this is used when all barcodes (or other sequences) that can be found in the experiment are known in advance. Users can decide whether to be error tolerant in the matching. By foregoing error tolerance, users achieve faster, linear-time processing at the expense of sensitivity. Alternatively, optimal aligners are employed, increasing sensitivity but decreasing speed. In the reference-free mode, barcodes are identified based on their structure as defined by the user. The structure of the barcode can be defined by conserved flanking regions on either side of the barcode, conserved base pairs in the barcode, or the combination of the two. This approach is used when the exact barcode sequences are not known in advance, for instance when the cells are randomly given a barcode from a pool of possible barcodes. QuiCAT integrates error correction mechanisms, combining NGRAMS-based pre-clustering and a forest-based refinement process. This approach efficiently groups and corrects barcodes for sequencing errors while pruning the number of pairwise comparisons. During barcode extraction, the pipeline tracks the associated sample. For scRNA-seq and SRT data, it also records the respective cell, spot, or spatial coordinates from which each barcode is derived, along with UMI information for UMI-based technologies. When working with BAM files as starting inputs, unlike other pipelines that start from unmapped reads, QuiCAT lifts this restriction, allowing users to specify any contig, transforming QuiCAT into a general tool for extracting any sequence of interest.

QuiCAT outputs a sample x cell x barcode or a sample x spot x barcode count matrix for scRNA-seq and SRT data, respectively. For population-based DNA data, the output is a sample x barcode count matrix. The matrices are stored in both AnnData and CSV formats, ensuring compatibility with Python-based analysis tools while allowing users to import data into other frameworks as needed.

Additionally, QuiCAT includes a simulation module capable of generating both bulkDNA and scRNA-seq synthetic datasets. Users can opt for a controlled setup maintaining full control over generated barcodes and their distributions, or an empirical simulation using state of the art sequencing simulators.

QuiCAT is a modular Python package featuring both a command line interface (CLI) and an application programming interface (API). The CLI handles tasks such as simulating and extracting synthetic tags from sequencing data, while the API allows users to import the results into an AnnData object (Virshup *et al*. 2024) and extends SCANPY’s (Wolf *et al*. 2018) plotting functionalities. Each CLI workflow accepts configuration files, allowing the user to specify runtime parameters.

### 2.2 Reads filtering

Users define filtering parameters, including a PHRED (Ewing *et al*. 1998) quality threshold and the minimum fraction of bases in a read that must meet this threshold. Since droplet-based scRNA-seq methods sequence all droplets, most of which are empty, users can provide a whitelist of cells, typically produced by alignment pipelines, ensuring that QuiCAT only scans reads from whitelisted cells, thereby further reducing runtime.

The pipeline filters out reads that fail to meet these criteria. It then passes the remaining reads to the barcode extraction steps, preserving cell or spot barcode information for scRNA-seq and SRT data. For UMI-based technologies the dominating barcode with highest frequency for each UMI is retained, limiting noise due to sequencing errors. Additionally, to remove low-count barcodes that may result from PCR artifacts, we include an optional pre-filtering step based on either the absolute count of each UMI–barcode combination or the relative abundance of the barcode in the entire dataset, which the user can activate.

### 2.3 Reference-based barcodes extraction

In the reference-based workflow (Supplementary Fig. 1, green section), users provide known barcode sequences as a reference, which can be supplied in different formats. Since modern sequencers exhibit a low probability of base-calling errors (Stoler and Nekrutenko 2021), QuiCAT allows the user to specify the allowed number of alignment mismatches in the input configuration file. When the tolerance is set to 0, QuiCAT employs the Aho-Corasick algorithm (Aho and Corasick 1975) to efficiently extract matching barcodes in linear time. If a tolerance value is specified, QuiCAT switches to the optimal aligner Edlib (Sosic and Sikic 2017), extracting barcodes in quadratic time.

### 2.4 Reference-free barcodes extraction

The reference-free workflow (Supplementary Fig. 1, red section) allows users to define known flanking regions of the barcodes—upstream, downstream, or both—and optionally specify a length interval for accepted sequences. Similar to the reference-based workflow, users can specify a mismatch tolerance for the flanking regions. If a mismatch tolerance is set in the input configuration files, QuiCAT uses CUTADAPT (Martin 2011) to extract barcodes. Otherwise, it uses regular expression matching (REGEX). Additionally, users can specify a masked pattern using the character “N” when part of the barcode’s internal structure is known, as in LARRY (Weinreb *et al*. 2020).

Once barcodes are extracted, users can enable sequencing error correction by specifying a distance threshold. If a threshold is provided, QuiCAT collapses low-frequency sequences into higher-frequency ones within the specified distance. This entails a two-step clustering process: first, a rapid pre-clustering using NGRAMS, followed by a more refined, tree-based method.

QuiCAT starts by building an NGRAMS matrix from the extracted sequences to create multiple fingerprints of each barcode. Following the Dirichlet Box principle, if the user wants to allow up to K mismatches we only need to split the barcodes in K + 1 fingerprints, thus we compute the NGRAMS’ length necessary to split the barcodes in K + 1 fingerprints using Equation (1), where L represents the barcode length and K is the distance threshold set by the user.


(1) n=L/(K+1)

The pipeline then iteratively multiplies vectors in the matrix to identify sequences sharing at least one NGRAM. It iteratively groups matching sequences into sets and removes them from the matrix, reducing the number of necessary multiplications. This process is repeated until the matrix is empty.

QuiCAT separately processes each set containing two or more sequences using a forest-based refinement step. It selects the barcode with the highest count as the root node and iteratively compares the others to the root nodes. For variable-length barcodes, QuiCAT calculates the Levenshtein distance (Levenshtein 1966)—while for fixed-length ones, it applies the Hamming distance (Hamming 1950). If a barcode lies within the specified threshold distance of a root node, QuiCAT assigns it as a child node. Otherwise, it initializes a new root node. By limiting comparisons to root nodes during each iteration, we reduce the number of pairwise comparisons and accelerates the process. This refinement continues until all barcodes are clustered. Given that the sets are disjoint, the process is parallelized. Each resulting tree forms a cluster, which QuiCAT then uses to merge barcode counts.

Unlike other pipelines that run STARCODE (Zorita *et al*. 2015) on each sample individually, QuiCAT performs clustering across the entire dataset, while applying count correction at the sample level. This is particularly beneficial in scRNA-seq datasets where sampling bias can occur due to the limited number of cells analyzed relative to the tissue of origin (Bonham-Carter and Schiebinger 2024). After identifying groups of barcodes within a specified collapsing distance threshold, the ones with lower counts are merged into those with higher counts. Users can also specify a barcode ratio, ensuring that barcodes with lower counts are collapsed only if the count of the major barcode is higher by a specified factor. By clustering globally, QuiCAT prevents the accidental collapse of real barcodes, even when they appear at low frequencies in some samples.

### 2.5 Barcode simulation

To support pipeline benchmarking and provide users with a tool for simulating barcoding libraries, we integrated a dedicated simulation workflow into QuiCAT. This workflow helps users fine-tune QuiCAT parameters before processing real data by generating synthetic datasets under two scenarios: controlled simulation and empirical simulation.

In the controlled simulation, users can specify the number of barcodes to simulate their length, the minimum Hamming distance between them, the flanking regions, and the distribution type. Supported distributions include uniform, random, normal, and power law, each with adjustable parameters. Users can also configure the number of PCR chimeras for each real barcode and control their relative abundance compared to the original barcodes. This process generates sequences with high quality scores and no sequencing errors in the flanking regions. This setup enables precise runtime comparisons across different pipelines.

For accuracy assessment, users can generate empirical simulations, modeling real sequencing conditions. In this workflow, QuiCAT generates a fixed number of real barcodes—following users’ specified properties—which are then stored in a FASTA file. These barcodes serve as input for the ART simulator (Huang *et al*. 2012). Beyond pipeline benchmarking, this approach allows users to explore the potential barcode space and refine experimental designs. The workflow will produce FASTQ files with the simulated barcodes along with a CSV file containing the ground truth real sequences in both scenarios.

## 3 Results

### 3.1 Quicat enhances performance in barcode extraction

To evaluate QuiCAT’s performance, we compared its barcode extraction efficiency with two recently published tools: BARTab and Pycashier. To extend the benchmark performed in the BARTab manuscript, we applied the three different pipelines to a publicly available dataset (Goyal *et al*. 2023), which encompasses population-level cellular DNA barcoding of 22 samples with 4 technical replicates each. We ensured consistency by applying similar parameters across all pipelines and limiting CPU usage to at most 20 cores.

**Figure 2.**
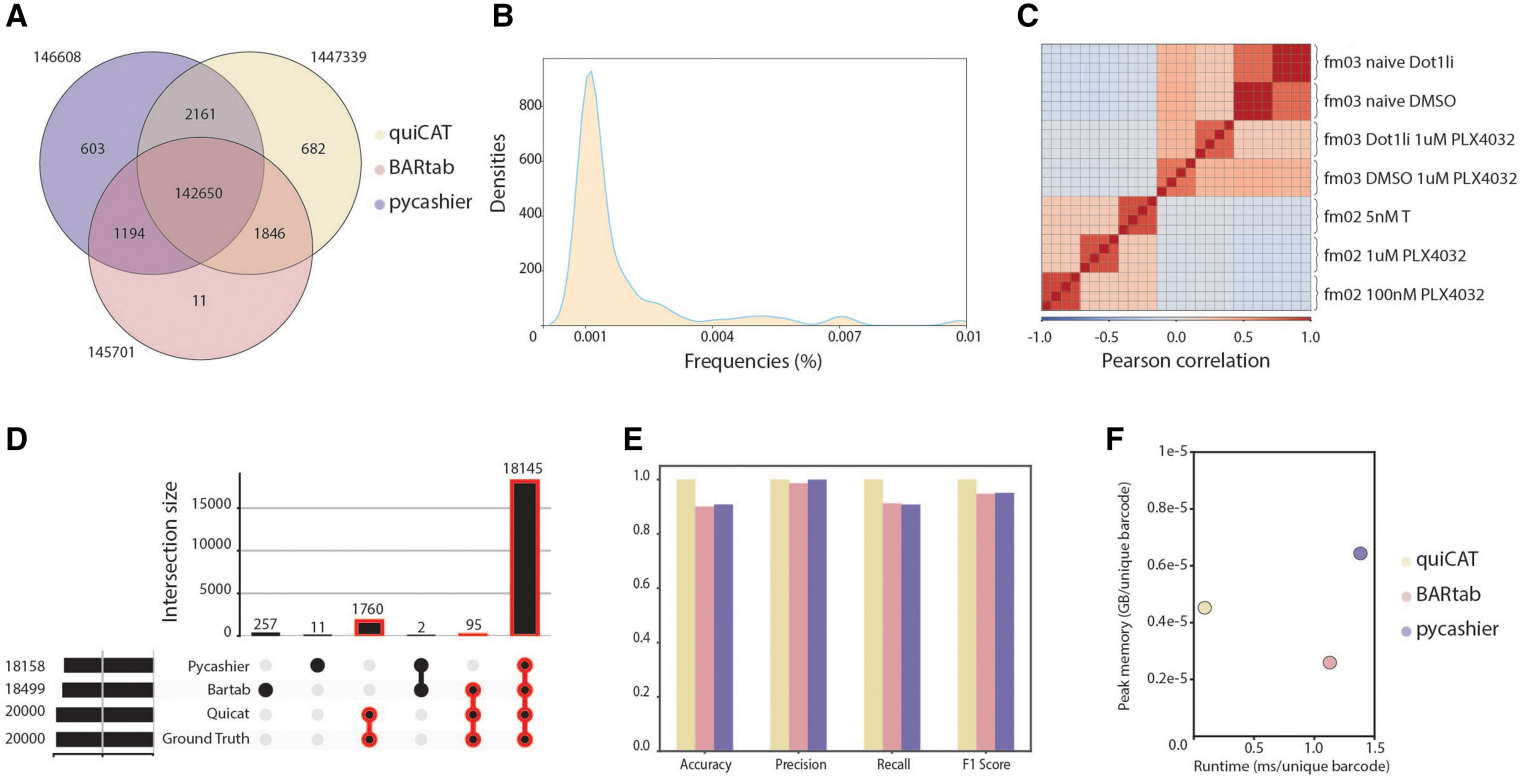
Benchmarking of QuiCAT against available barcode-extraction pipelines displays its advantages. (A) The three tested pipelines show a high overlap in detected barcode sequences in a real bulk DNA sequencing dataset (Goyal et al. 2023). (B) The frequency distribution of barcodes that are not detected by all three pipelines shows that they predominantly occur at low frequencies, near the filtering threshold. (C) The correlation matrix on a subset of samples shows a high correlation between the four technical replicates (indicated by brackets) in the QuiCAT output, demonstrating robustness. Melanoma cell line WM989 was either pretreated with DMSO or DOT1Li, followed by treatment with the BRAF inhibitor vemurafenib (PLX4032, 1 µM)—fm03; or was treated with 1 µM or 100 nM PLX4032 or MEK-inhibition with 5 nM trametinib (T) without pretreatment—fm02. (D) Results on a synthetic dataset of 20 000 barcodes generated with QuiCAT demonstrate superior barcode detection, with a 100% match to the ground truth for QuiCAT, compared to the two other pipelines. (E) Accuracy, precision, recall, and F1 score of the benchmarked tools on the synthetic dataset. (F) Averaged Peak memory usage (reported as GB per unique barcode) and runtime (reported as milliseconds per unique barcode) on the synthetic and Goyal dataset extraction workflows.

We observed 142 650 (95.7%) sequences with at least 0.001% frequencies detected by all three tools (Fig. 2A). An additional 1846 sequences (1.2%) were only identified by BARTab and QuiCAT due to Pycashier’s restricted length flexibility. The remaining non-overlapping barcodes, those identified by only one or two of the tools, are predominantly found near the frequency filtering threshold (Fig. 2B). This difference likely arises from QuiCAT’s use of a global clustering approach, as opposed to the individual clustering strategies used by the other two pipelines. To assess QuiCAT’s robustness we computed pearson correlations between technical replicates on a subset of samples. Briefly, the melanoma cell line WM989 was split into two treatment groups. Group fm03 was pretreated for 5 days with either DMSO or 4 µM DOT1L inhibitor (Dot1li), followed by 1uM treatment with B-RafV600E inhibitor vemurafenib (PLX4032). Group fm02 was directly treated with 1 µM or 100 nM vemurafenib or 5 nM trametinib. QuiCAT barcode extraction yielded strong correlation between technical replicates and between samples belonging to the same treatment group, demonstrating its robustness (Fig. 2C).

Since the Goyal dataset lacks a ground truth, we next assessed the performances of the three pipelines on a synthetic dataset generated with QuiCAT to verify accuracy under controlled conditions. The dataset contains four samples, each containing 5000 (60 bp long) sequences, with a minimum Hamming distance of 6 and fixed flanking regions. Once generated, these barcodes served as input for the ART simulator to produce 2000 reads per barcode in amplicon mode. During barcode extraction, all three pipelines were run with comparable parameters. BARTab and Pycashier failed to recapitulate the set of barcodes in the ground truth by detecting significantly fewer barcodes and additionally producing false positives, whereas QuiCAT correctly reconstructed the ground truth set of barcodes with no false positives (Fig. 2D and E). QuiCAT’s accurate reconstruction underscores its robustness and analytical precision, which is critical in applications demanding high-fidelity barcode identification and quantification such as including lineage tracing and CRISPR-based genomic screens.

Beyond accuracy, we assessed the computational performances of the three pipelines. In terms of speed, QuiCAT outperformed the other tools in both datasets with up to a 13-fold improvement in runtime compared to the second-best performing tool in both the Goyal and synthetic datasets (Fig. 2F). QuiCAT’s speed advantage primarily results from its optimized barcode collapsing and error correction algorithms, with the benefits of this approach becoming more apparent as the library complexity increases and more closely resembles real-world datasets. Furthermore, QuiCAT maintained low peak memory consumption despite avoiding the storage of intermediate files (Fig. 2F).

To extend the benchmark on additional real-world data, we created an in-house dataset with available ground truth. Briefly, 40 individual clones were isolated from a PDAC cell line and simultaneously individually barcoded with a modified version of LARRY (Weinreb *et al*. 2020) with a 16 bp barcode followed by a conserved region (Supplementary material). The barcode of each clone was independently confirmed by sequencing (Supplementary Fig. 2A). Then, the clones were pooled in equal amounts, expanded for 24 hours, and the barcodes were amplified and sequenced in a pooled fashion (Supplementary Fig. 2A). For benchmark purposes, the barcodes of the pooled sample were detected using the reference-free workflows of each tool—Pycashier, Bartab and QuiCAT—with comparable parameters. In each case, the barcodes’ correction was enabled allowing up to two mismatched nucleotides. Using this setup QuiCAT successfully extracted all forty expected barcodes, with one detected false positive barcode, representing 0.6% of relative abundance (Supplementary Fig. 2B), resulting in the best overall performance (Supplementary Fig. 2E). Similarly, Pycashier retrieved all 40 expected barcodes but with three false positives, while Bartab only detected 33 barcodes and eighteen false positives (Supplementary Fig. 2D), resulting in the worst performance overall (Supplementary Fig. 2E). To further assess QuiCAT capabilities, we compared the reference-free to reference-based extraction. In the reference-based extraction, the barcode sequences extracted from the individual clone sequencing were used as input reference, allowing up to two mismatches in the alignment to match the two allowed mismatches in the sequencing error correction of the reference-free setup. Among the expected barcodes we found a 100% match in barcode counts, between the reference-free and reference-based extraction workflow (Supplementary Fig. 2C). While Pycashier does not offer a reference-based method, we attempted to evaluate QuiCAT against BARTab. However, BARTab repeatedly failed to extract the barcodes. Its reliance on Bowtie1 (Langmead *et al*. 2009), optimized for aligning short reads to long reference genomes, proved unsuitable for barcode extraction where references are often shorter than reads. Even when references were masked with “N” positions to artificially extend their length, BARTab was unable to complete a successful extraction run. Building upon QuiCAT’s demonstrated efficiency and accuracy in barcode extraction, we next evaluated its adaptability across various sequencing technologies and barcode libraries to show how its high degree of customizability can accommodate a variety of experimental designs. All the analyses and benchmarks were executed on an on-premises server with 378 GB of available RAM and two Intel Xeon Gold 6230 CPUs, providing a total of forty cores and eighty threads.

### 3.2 Reference-based extraction of combinatorial barcodes in single-cell RNA sequencing

Expressed cellular barcoding systems paired with single cell readouts enable the tracking of clonal populations and associated transcriptomic changes across different conditions, such as genetic or therapeutic perturbations. To demonstrate QuiCAT’s versatility, we applied its reference-based workflow to capture and analyze expressed barcodes in single-cell RNA sequencing data.

We performed an in vitro experiment using Pro-code barcodes—unique combinations of short protein encoding tags fused to the mCherry reporter gene (Dhainaut *et al*. 2022). The expressed barcodes are typically detected using antibody-based techniques. However, to simultaneously obtain the single-cell transcriptomes, we opted for mRNA-based barcode detection.

**Figure 3.**
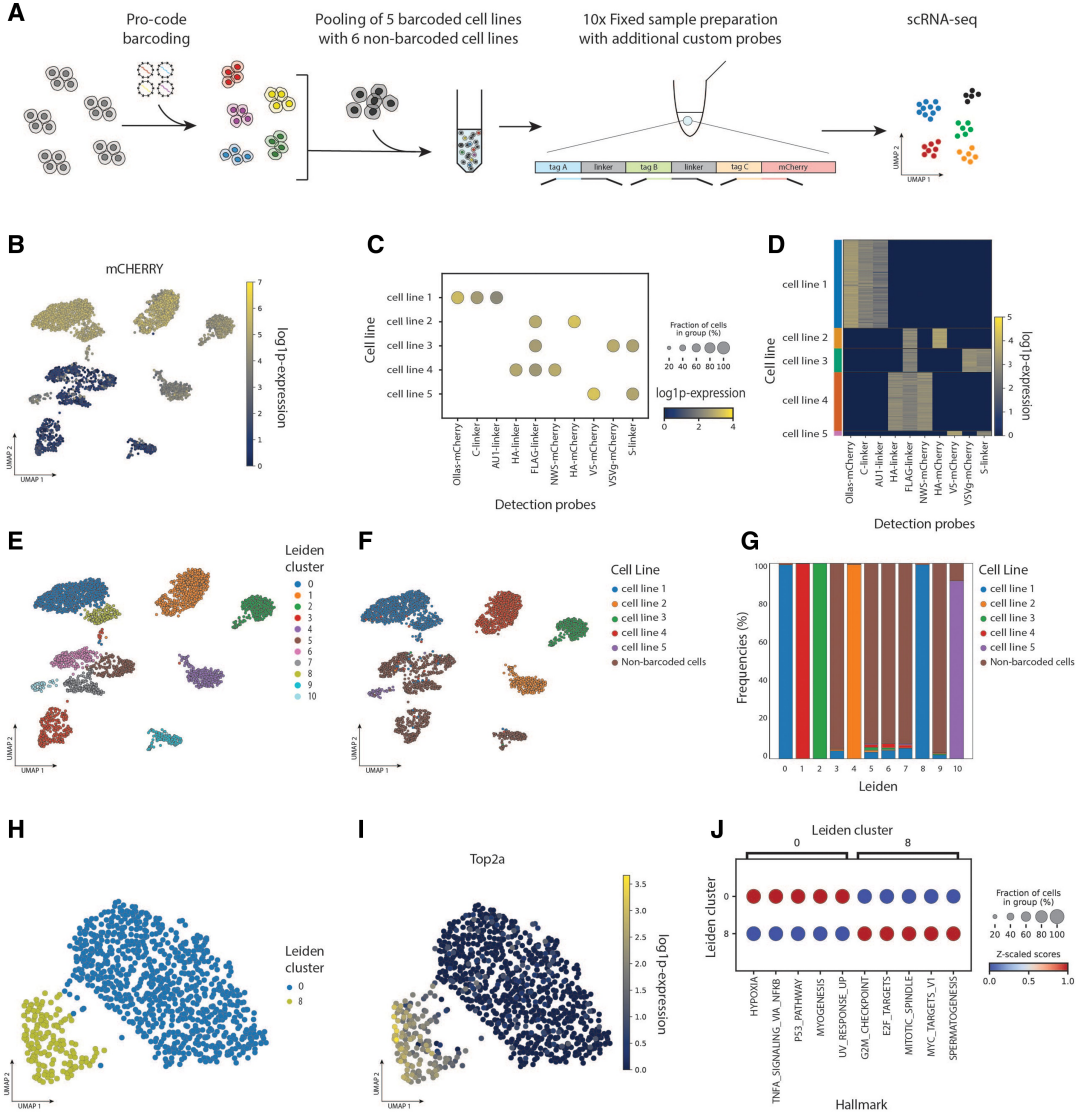
QuiCAT demonstrated reference-based barcode extraction and analysis of the in-house scRNA-seq. (A) Experimental design overview. A Pro-code with 3 Pro-code tags was used to barcode each cell line and was pooled with non-barcoded cell lines prior to library preparation and sequencing. (B) mCherry+ barcoded cells cluster apart from non-barcoded cell lines. (C, D) The cell lines were assigned based on mCherry expression status and expected combination of two or three Pro-code tags. (E) Leiden clustering based on gene expression identified 11 distinct clusters. (F) Leiden clusters overlap with cell lines based on Pro-code tag expression. (G) Representation of cell lines across Leiden clusters shows that different cell lines tend to segregate transcriptionally with high concordance of Leiden clusters and different barcoded cells. (H, I) UMAP of cell line 1 coloured by Leiden clusters (H), and expression of the proliferation marker gene Top2a (I), which is increased in cluster 8. (J) Gene enrichment analysis shows Leiden cluster 8 to express Hallmark gene sets associated with cell cycle progression and increased proliferation.

Five pancreatic ductal adenocarcinoma (PDAC) murine clonal cell lines were individually tagged with unique combinations of 2–3 Pro-code barcodes. After isolating one PDAC clone per cell line, we verified the barcodes using Sanger sequencing and added six additional non-barcoded PDAC murine cell lines. Since the barcodes vary in total length, with some barcode combinations exceeding the most commonly used scRNA-seq read lengths, we selected a probe-based hybridization approach for combinatorial barcode detection with 10x Chromium Single Cell Gene Expression Flex kit. To detect mCherry and Pro-code tags, we designed specific hybridization probes for each of the Pro-codes and mCherry (Fig. 3A) (detailed methods in Supplementary Material).

Using three probes, as recommended by the Flex protocol, we successfully retrieved mCherry sequences with 10x Genomics Cell Ranger v7.2.0. However, it was not feasible to design three probes for the Pro-code tags because of their limited lengths. Therefore, only one probe per barcode tag was designed. Given the Pro-code tags’ length variability and absence of a shared flanking region, we employed QuiCAT’s reference-based workflow for targeted extraction.

After applying filtering steps on the gene expression library to exclude cells with detected transcripts/genes outside two median absolute deviations according to current single-cell best practices (Heumos *et al*. 2023), after the filtering we were left with 5930 high quality cells (59.7% of the dataset). Next we integrated the QuiCAT output with the gene expression AnnData object. Positive mCherry barcoded cell lines notably cluster apart from the non-barcoded cell lines (Fig. 3B). We then assigned cells to clones using QuiCAT’s API by comparing the set of detected barcodes per cell against the expected Pro-code tag combinations. Since our library included clones carrying combinations of two or three barcodes, the assignment procedure evaluated which predefined combination best matched the observed barcodes. To ensure robust assignments, cells were only mapped to a clone if the best match reached at least 75% similarity with the expected barcode combinations (Fig. 3C and D). The 75% cutoff was chosen as a balance between sensitivity and specificity reducing the risk of misassigning cells due to barcode dropout or spurious detection, while at the same time avoiding the exclusion of a large fraction of true clonal cells affected by imperfect barcode capture. Empirically, this threshold provided a practical compromise, allowing us to assign a clone to every Pro-code positive cell.

To explore differences among the cell lines, we identified 11 Leiden clusters based on gene expression (Fig. 3E). Cell line assignments were mapped onto these clusters, revealing strong concordance (Fig. 3G). Interestingly, cell line 1 appeared in two separate Leiden clusters, both linked to the same starting cell clone of the cell line through synthetic barcoding. A closer examination showed distinct transcription profiles between these two clusters, indicating plasticity or tumor evolution of the cells over the prolonged culturing period during clonal isolation (Fig. 3H and I). Gene enrichment analysis performed with Decoupler (Badia-I-Mompel *et al*. 2022) and Liana (Dimitrov *et al*. 2024) revealed increased proliferative activity in cluster 8 (Fig. 3J).

Having validated QuiCAT’s ability to accurately extract Pro-code barcodes and integrate them with transcriptomic data, we next sought to compare its performance against Bartab. As for the in-house dataset from the previous section, we were unfortunately unable to complete a successful run using Bartab reference-based workflow for performance comparison due to the limitations of the tool.

### 3.3 Quicat enables barcode extraction in spatial transcriptomics

Spatial transcriptomics has transformed transcriptome analysis by incorporating spatial information, allowing researchers to investigate cellular interactions and neighborhoods. Most techniques, including Visium and Stereo-seq, utilise fixed barcode-labeled-oligonucleotides to link the mRNA transcript to the spatial coordinates.

To demonstrate barcode extraction in a spatial dataset, we applied QuiCAT to a study exploring clonal relationships in the developing mouse brain (Ratz *et al*. 2022). Briefly, mouse embryonic progenitor cells were labeled in vivo on embryonic day 9.5 using a lentiviral library, incorporating a 30 bp random barcode sequence downstream of the nuclear-localized EGFP. The mice were sacrificed and analyzed at around postnatal day P14. Visium spatial transcriptomics and immunostaining were performed on eight consecutive 10 µm brain sections from one mouse.

**Figure 4.**
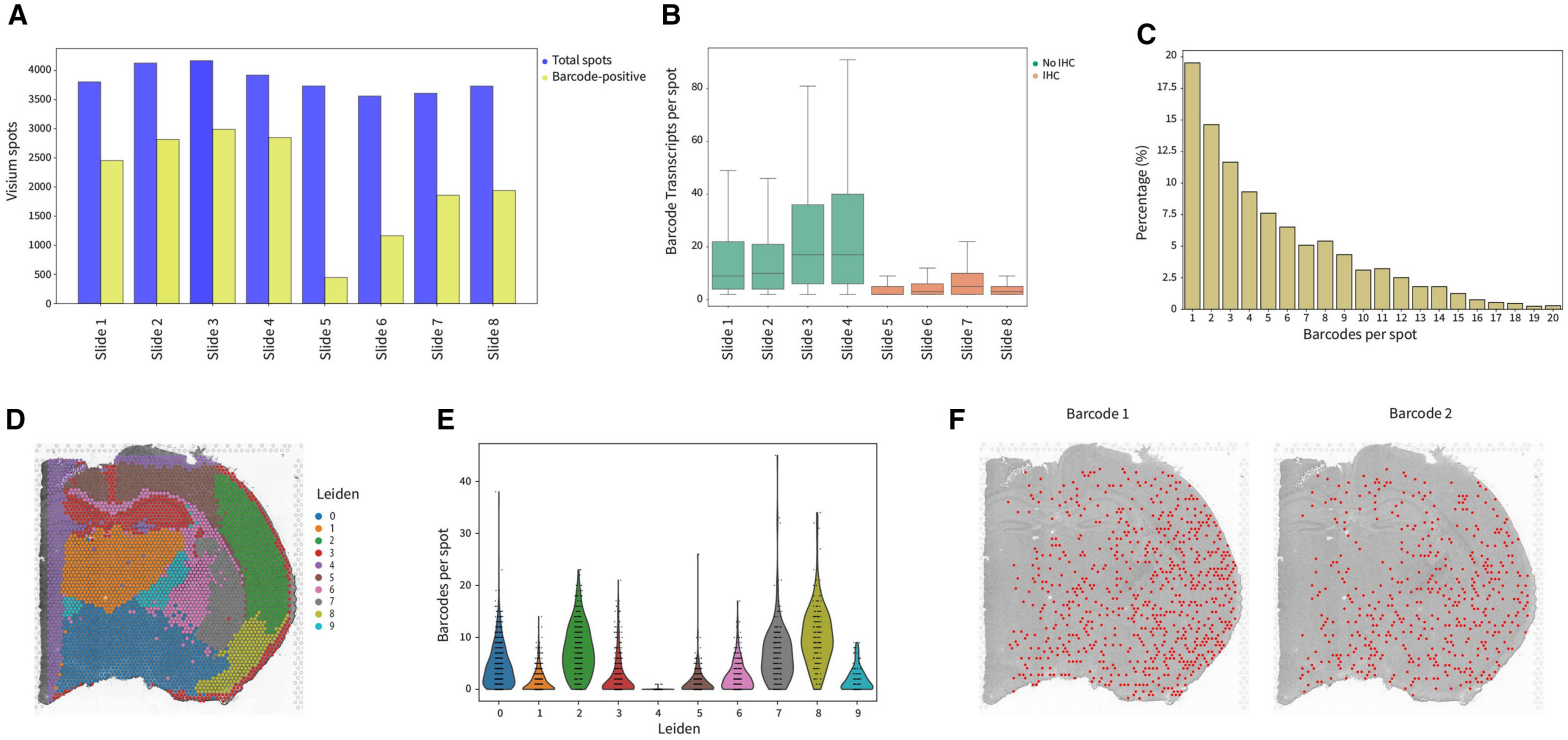
Barcodes can be extracted from spatial transcriptomic data with QuiCAT. Briefly, mouse embryonic progenitor cells were labeled with expressed barcodes in vivo on embryonic day 9.5 and the brain was sampled around postnatal day P14. 10x Visium spatial transcriptomics was performed on eight consecutive brain sections. (A) Barcode-positive Visium spots compared to the total number of spots in the eight different Visium samples. (B) Fewer barcodes were retrieved in samples that underwent IHC prior to the Visium run indicating lower quality of the slides #5–8. (C) Detected number of different barcodes per spot combining all slides. (D) Gene expression-based Leiden clustering on slide #1. (E) Barcodes per spot show variability across clusters, with clusters 2, 7, and 8 exhibiting the highest barcode counts. (F) The two most abundant barcodes are spatially distributed over all brain regions.

Using QuiCAT in reference-free mode, we extracted barcodes from all eight Visium slides using FASTQ reads. Barcode presence varied substantially between slides (Fig. 4A). Notably, slides 1–4 exhibited higher endogenous transcripts and barcode detection compared to slides 5–8, likely due to immunohistochemistry (IHC) staining performed prior to Visium processing in the latter group (Fig. 4B) as described in the original publication. Notably, barcode detection in slide 5 was lower than in all other IHC-stained slides, in line with lower overall transcript abundance in this sample. In general, most spots contained between one and five barcodes (Fig. 4C). Focusing the analysis on slide 1, we performed Leiden clustering based on transcriptomic profile, which delineated distinct brain regions, consistent with the original publication (Fig. 4D). Next, we examined the abundance of different barcodes among Leiden clusters and observed higher barcode variability in clusters 2, 7, and 8 (Fig. 4E). Almost no barcodes were detected in cluster 4 due to its position at the edge of the slide, which reduced spots qualities and the number of detected transcripts and barcodes overall. In general, we observe a correlation between the detection of endogenous transcripts and barcodes. To investigate clonal localization patterns, we focused on the two most abundant clones and generally observed a lack of spatial segregation (Fig. 4F). This widespread barcode distribution aligns with expectations since barcoding happened early in brain development (E9.5). As a result, the barcode was passed on to progeny populating all investigated brain regions.

### 3.4. Extraction of sgRNA coupled to scRNA-seq on Perturb seq dataset

**Figure 5.**
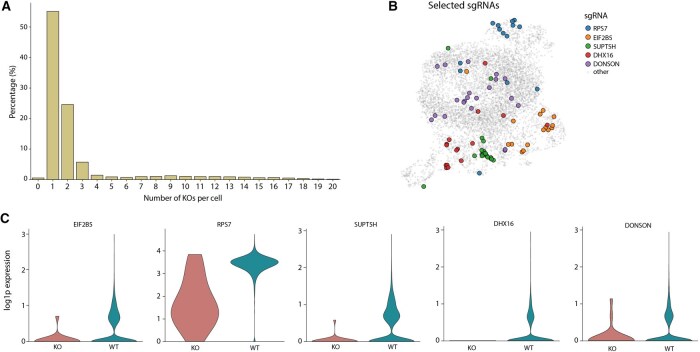
QuiCAT enables extraction of sgRNAs from Perturb-seq datasets. sgRNAs were extracted from a single sample targeting 2203 essential genes. (A) Most cells contained a single sgRNA, consistent with expectations. (B) Cells carrying different perturbations showed distinct localization in the UMAP embedding. (C) Targeted genes’ expression was markedly reduced in cells containing the corresponding sgRNA compared to other cells.

Recent advances in single-cell methods, such as Perturb-seq, allow for the coupling of CRISPR screens with scRNA-seq (Dixit *et al*. 2016). To demonstrate QuiCAT’s utility beyond cellular barcodes, we applied it to a single sample (KD6_1_essential) of a public Perturb-seq dataset (Replogle *et al*. 2022) targeting 2203 essential genes. For efficient gene depletion, the study used two single-guided RNAs (sgRNAs) targeting the same gene that were both present in the same construct (dual-sgRNA construct) and were therefore expressed in the same cells. For demonstration purposes we limited the analysis to a single sample from the study. Using the reference-based extraction workflow of QuiCAT, we extracted the sgRNAs and mapped them to their origin cell. We found that most cells contained a single sgRNA pair, while a smaller fraction contained multiple pairs (Fig. 5A). After quality control and preprocessing according to single-cell best practices (Heumos *et al*. 2023), we found that cells containing identical sgRNAs frequently appeared close together in the UMAP embedding (Fig. 5B). As expected, the expression of a targeted gene was substantially reduced in cells containing the corresponding sgRNA, confirming both the efficacy of the screen and the accuracy of our extraction.

## 4 Discussion

In this work, we introduced QuiCAT, an end-to-end Python package for the analysis of synthetic DNA and RNA barcodes. Benchmarking demonstrated QuiCAT’s ability to extract barcodes across different datasets, outperforming current pipelines in both accuracy and speed. QuiCAT’s reference-based alignment strategy demonstrated superior flexibility in sequence retrieval compared to existing pipelines. Those pipelines either lack reference-based support entirely or rely on general-purpose aligners, which are often suboptimal for barcode extraction, with Bartab repeatedly failing to complete a successful run when a reference is provided, while Pycashier entirely lacking the reference-based workflow. Additionally, by leveraging optimized algorithms in both the reference-free and reference-based approaches, QuiCAT ensures robust detection of barcodes while dramatically increasing computational performances. This is particularly important in emerging high-throughput applications where scalability and accuracy are crucial.

QuiCAT’s modular and open-source design, combined with user-configurable parameters for fine-grained control, grants flexibility across different datasets, and various synthetic cellular barcoding systems. At the current stage, QuiCAT supports barcode extraction from DNA sequencing data in both single-end and paired-end formats, 10x Genomics datasets including Chromium, Flex, and Visium, spatial datasets generated with Stereoseq, as well as single-cell datasets from Parse Biosciences, as these are the most prominent in recent literature. Additionally, given the modularity of QuiCAT’s code, adding support for emerging technologies will be fast and easy, making the pipeline future-proof. Moreover, QuiCAT outputs are compatible with Scanpy for downstream tasks by directly outputting an AnnData H5AD file. Alternatively, QuiCAT’s CSV output can be imported into any framework of the user’s choice, ensuring interoperability.

QuiCAT accepts both FASTQ and BAM inputs. When working with FASTQ files, every read in the dataset is scanned for sequences of interest. In single-cell or spatial transcriptomics datasets, where alignment is typically performed with the vendor-provided pipelines, users can instead use the resulting BAM files as the starting point. Unlike other pipelines that restrict analysis to unmapped reads when working with BAM files, QuiCAT relaxes this constraint by allowing users to specify whether to focus on reads mapped to a given contig, to use only unmapped reads, or to process the entire read set. This feature enables targeted extraction of known variants of interest, removing the rigid constraints imposed by existing barcode extraction tools. Consequently, QuiCAT has the theoretical potential to function as a general-purpose sequence extractor, extending its usability to a broader range of applications beyond synthetic barcoding. We showcase this increased flexibility in a public Perturb-seq dataset using QuiCAT to extract sgRNAs and map them to their cell of origin. However, it is important to note that while QuiCAT’s flexibility theoretically allows it to retrieve sequences of interest in different contexts, it has not been explicitly tested for use cases beyond barcode extraction and Perturb-seq datasets. Users exploring novel applications should carefully validate performance for their specific needs where additional optimization may be required. For example, QuiCAT has not yet been specifically tested for dynamic barcoding systems. Most dynamic barcoding systems employ a two-barcode structure with one dynamic and one static barcode, such as macsGESTALT (Simeonov *et al*. 2021). In this scenario the user could theoretically run the extraction workflow twice and combine the two outputs afterwards. For dynamic systems that rely on a single barcode like LINNAEUS (Spanjaard *et al*. 2018), QuiCAT’s reference-free workflow could potentially be utilised, but users would need to carefully adjust the parameters, especially when performing sequencing error correction. These improvements could be implemented in future iterations if demand for these use cases arises.

Additionally, while QuiCAT demonstrated strong performance in benchmarking tests without a significant increase in memory footprint compared to other pipelines, users should still be aware that the QuiCAT processes all data in memory. This architecture enables fast execution but may lead to high random-access memory (RAM) usage for extremely large datasets, particularly through the creation of the NGRAMS matrix. In cases where barcode libraries are highly complex, users may need to monitor system memory availability and adjust computational resources accordingly. Future iterations of QuiCAT may explore memory-efficient strategies, such as chunked processing, if memory footprint becomes a bottleneck.

## Supplementary Material

btaf607_Supplementary_Data

## Data Availability

The bulk DNA dataset is available at Figshare (https://doi.org/10.6084/m9.figshare.22806494). The scRNA-seq dataset, and the LARRY clonal cell lines DNA dataset generated in this study are publicly available at Zenodo (https://doi.org/10.5281/zenodo.15063941). The spatial dataset is available in Gene Expression Omnibus under accession code GSE153424. The Perturb-seq dataset can be found at https://gwps.wi.mit.edu/.

## Code availability statement

QuiCAT is available to be installed as a python package. The source code is publicly available and can be found at https://github.com/theislab/quicat. The code to reproduce the analyses in the manuscript together with the version of QuiCAT used in the paper can be found at https://doi.org/10.5281/zenodo.15063941.
