# Supplementary material for: QuiCAT: a scalable and flexible framework for mapping synthetic sequences
Source: Bioinformatics. 2025 Nov 11;41(12):btaf607. doi: 10.1093/bioinformatics/btaf607 (PMC12684725; doi:10.1093/bioinformatics/btaf607)
Supplement: btaf607_Supplementary_Data [file btaf607_supplementary_data.zip › 17-Nov-2025_105002_QuiCAT_supplementary_data-FINAL.docx]

# **Supplementary material**

### Production of lentivirus-containing medium

HEK293 T cells were grown in 6-well plates for production of Pro-code lentiviruses. At 60-70 % confluency the cells were transfected using Lipofectamine 3000 transfection reagent according to the manufacturer's protocol. Transfection mix contained packaging plasmids psPAX2 (Addgene #12260; 800 ng per reaction) and pMD2G (Addgene #12259; 300 ng per reaction), and individual plasmid from Nuclear procode vector kit library (Addgene #1000000197; 1000 ng per reaction) (Dhainaut 2022). After 24 h the medium was changed to DMEM (Sigma-Aldrich, St. Louis, USA) with 1 % PenStrep (Sigma-Aldrich, St. Louis, USA) and 20 % FCS (Sigma-Aldrich, St. Louis, USA), and the following two days, the medium was harvested, filtered through 0.45 μm filter, and stored at -80 °C. The Nuclear Pro-Code Vector Kit was a gift from Brian Brown (Addgene kit #1000000197).

### Barcoding cell lines with Pro-codes

Five cell lines were plated separately in a 6-well cell culture plate at density 100000 cells per well. The following day, the cells were transduced by adding 200 ul of lentivirus-containing media of individual pro-code combination, and polybrene to final concentration 8 ng/ml. After 24 h, the cells were trypsinized and plated in a serial dilution in a 96-well plate to generate clonal cell lines. Clonal cell lines showing positive mCherry signal were expanded and barcode sequences were confirmed with Sanger sequencing.

### PCR amplification and Sanger sequencing of Pro-code barcodes from clonal cell lines

### Cell pellets of clonal cell lines were lysed and the Pro-code region of DNA was amplified with PCR using primers Pro-Code_for2 (GTGTCGTGAGGAATTCTGCAGTC) and Pro-Code-rev (GTCACCTTCAGCTTGGCGG). PCR products were purified using Monarch® PCR & DNA Cleanup Kit (New England BioLabs Inc.). Purified amplicons were adjusted to concentration 5 ng/μL and sent to Sanger sequencing at Eurofins Genomics GmbH (Ebersberg).

### Cell line pooling, fixation, and library generation and sequencing

Eleven primary mouse PDAC cell lines, including five clonal cell lines with Pro-codes were cultured separately. The cells were trypsinized and pooled together in equal amounts, then 5 million cells were fixed according to the protocol (10x genomics, User guide CG000478). After fixation, a pool of 1 million cells was used for hybridization with probes, including custom probes, which lasted 19 h (10x genomics, User guide CG000691). For generation of GEM, 20000 cells were loaded on the chip and library was constructed as described in the guidelines (10x genomics,User guide CG000691). The sample was sequenced with Novaseq sequencer at depth of total 300 million reads with expected number of 10,000 cells.

### Custom probes for dete**ction of Pro-code barcodes and mChe**rry by Chromium single cell gene expression Flex

Custom probes were designed according to the Technical note CG000621 (10x Genomics). The sequences of custom probes are provided in Table 1. Three probes were designed for mCherry. For detection of Pro-code barcodes, left-hand-side probe parts were designed for linker region or for mCherry region, and the right-hand-side probe parts were designed for individual tags, so that all possible pairings of tags and either linker or mCherry are possible. The probes were used in recommended concentration and hybridized for 19 hours.

**Table 1** Custom probes and respective sequences for detection of Pro-code barcodes and mCherry with Chromium single cell gene expression Flex.

| robe-ID | sequence |
| --- | --- |
| LHS-Linker | ccttggcacccgagaattccaccctcccccgcctcctcc |
| LHS-mCherry | ccttggcacccgagaattccacttgctcaccatcagcatgcc |
| LHS-mCherry-tdTom-1 | ccttggcacccgagaattccaccgtcctcgaagttcatcacgcgct |
| LHS-mCherry-tdTom-2 | ccttggcacccgagaattccaggaggcctcccagcccatggtctt |
| LHS-mCherry-tdTom-3 | ccttggcacccgagaattccatcgtactgttccacgatggtgtagt |
| RHS-Flex-AU1 | /5phos/aatgtaccgataggtgtcgccacgcggttagcacgtannactttaggcggtcctagcaa |
| RHS-Flex-C | /5phos/cttgccgtcgatcagcctgggacgcggttagcacgtannactttaggcggtcctagcaa |
| RHS-Flex-FLAG | /5phos/cttgtcgtcatcgtctttgtaacgcggttagcacgtannactttaggcggtcctagcaa |
| RHS-Flex-HA | /5phos/agcgtaatctggcacatcgtaacgcggttagcacgtannactttaggcggtcctagcaa |
| RHS-Flex-HSV | /5phos/gtcctcggggtcctcgggggcacgcggttagcacgtannactttaggcggtcctagcaa |
| RHS-Flex-mCherry-tdTom-1 | /5phos/cccacttgaagccctcggggaaggaacgcggttagcacgtannactttaggcggtcctagcaa |
| RHS-Flex-mCherry-tdTom-2 | /5phos/cttctgcattacggggccgtcggacgcggttagcacgtannactttaggcggtcctagcaa |
| RHS-Flex-mCherry-tdTom-3 | /5phos/cctcgttgtgggaggtgatgtccaacgcggttagcacgtannactttaggcggtcctagcaa |
| RHS-Flex-NWS | /5phos/tttctcgaattgggggtgggaacgcggttagcacgtannactttaggcggtcctagcaa |
| RHS-Flex-Ollas | /5phos/gcacttgcccatcagcctgggacgcggttagcacgtannactttaggcggtcctagcaa |
| RHS-Flex-S | /5Phos/gctgtccatgtgctgcctctcACGCGGTTAGCACGTANNACTTTAGGCGGTCCTAGCAA |
| RHS-Flex-V5 | /5Phos/tgtggaatccagtcccagcagACGCGGTTAGCACGTANNACTTTAGGCGGTCCTAGCAA |
| RHS-Flex-VSVg | /5Phos/ctttccgagtctattcatttcACGCGGTTAGCACGTANNACTTTAGGCGGTCCTAGCAA |

### Detailed flow-diagram of the quiCAT pipeline


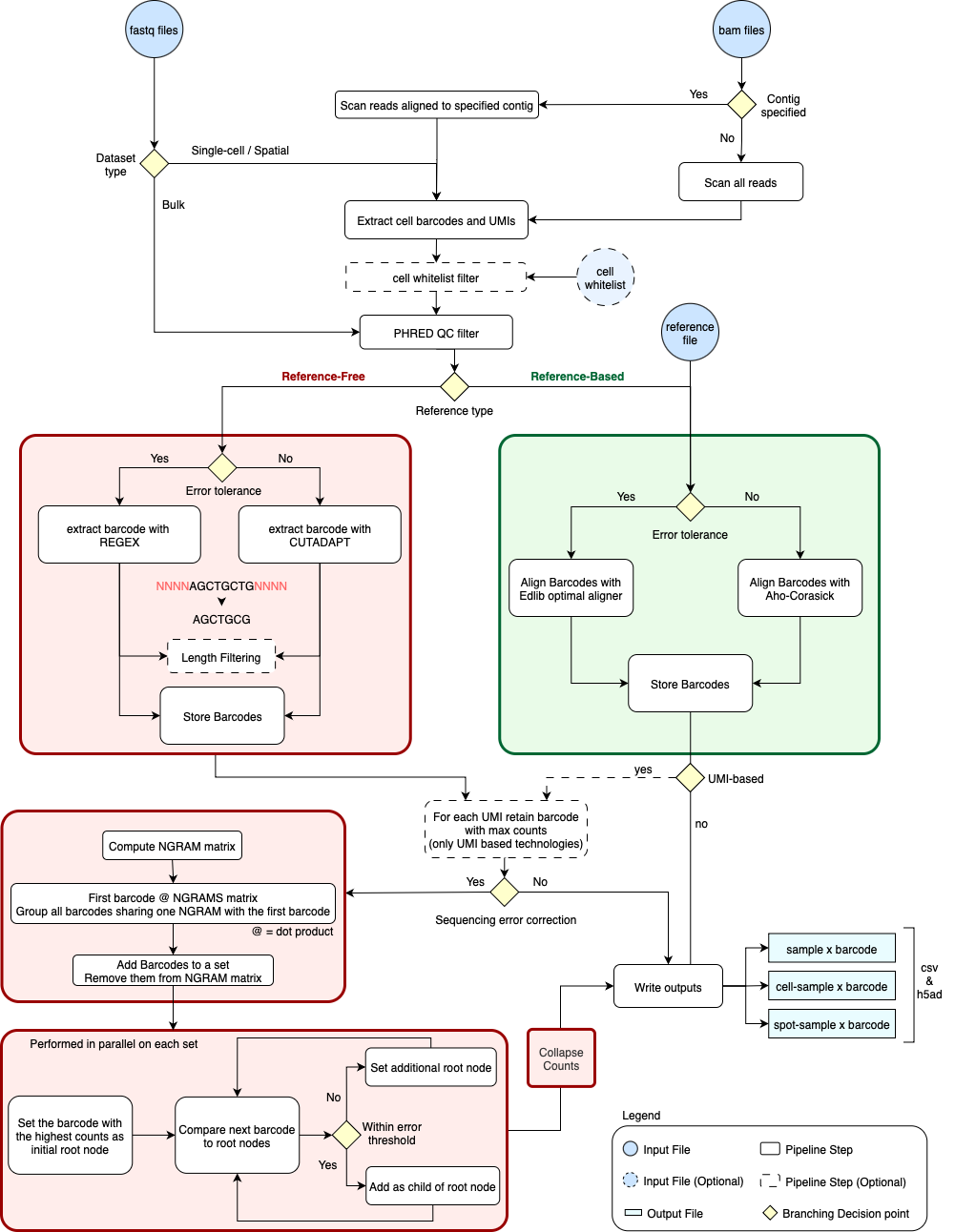


**Supplementary Figure 1**: Flow diagram of QuiCAT pipeline for reference-free (red) and reference-based (green) barcode extraction.

**Ground truth assessment of barcode recovery from pooled clonal cell lines**

40 unique lineage and RNA recovery (LARRY) DNA barcodes were generated using a modified LARRY barcoding system (Weinreb 2020; Addgene plasmid #140025). This modified construct includes the addition of a puromycin resistance marker, which is connected via a T2A self-cleaving peptide to the EGFP reporter containing a semi-random 16-mer in its 3’UTR. (sequence structure NNNNCTNNNNACNNNN). Lentiviral particles were produced for each unique pLARRY barcode as previously described.

To generate barcoded clones, one cell line was plated in 40 wells of a 24- well cell culture plate at a density of 100000 cells per well. The following day, each well was transduced with 500 uL of media containing one of the pLARRY barcodes, and 8 ng/mL Polybrene (Sigma-Aldrich, St. Louis, USA). Transduction was facilitated via spinfection at 33°C and 1000 x g, 33°C for 2 hours. 48 h post-transduction, 4 ug/mL puromycin (Merck, Dermstatt, Germany) was added to media for selection. After 48 h of selection, cells were serially diluted into 96-well plates to derive single-cell clones. GFP-positive clonal lines were expanded under continued puromycin selection. Barcode sequences from each clone were verified by NGS. Equal numbers of cells from each of the 40 verified clones were pooled in equal proportions, expanded for 24 hours and then immediately processed for barcode amplification. PCR was performed using primers targeting the flanking regions of the pLARRY barcode (forward: CAAGCAGAAGACGGCATACGAGATTAGCGAGTCTGAGCAAAGACCCCAACGAGAA, reverse: AATGATACGGCGACCACCGAGATCTACACTCGACTAGGAAGGCACAGGTCGACACCAGTCT)

PCR product was purified using Monarch® PCR & DNA Cleanup Kit (New England BioLabs Inc.) and subsequently sequenced using Illumina NGS.


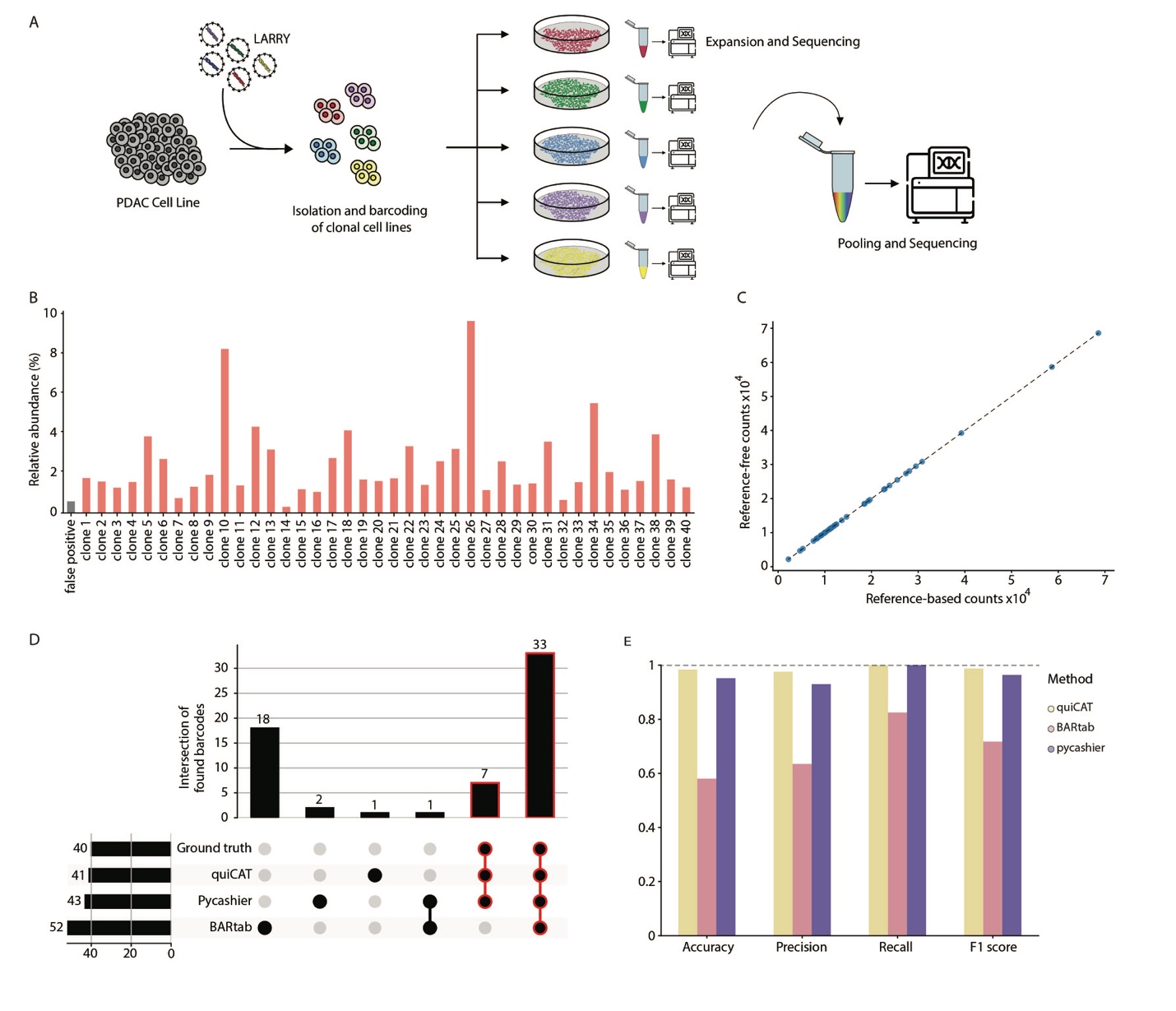


**Supplementary Figure 2**: Comparison of different extraction methods on an in-house DNA dataset. **A**) Overview of experimental design showing isolation and barcoding of clonal cell lines, followed by sequencing of barcode for each clone individually or for pooled clones. **B**) Relative abundance of individually barcoded clones extracted with QuiCAT using reference-free extraction. **C**) Comparison of barcodes’ absolute frequencies as extracted from the reference-free and reference-based QuiCAT workflows. **D**) Reference-free barcodes extraction workflows of QuiCAT, Pycashier and BARtab were benchmarked, with QuiCAT showing the lowest false positive rate. All the pipelines were run with error correction turned on, allowing up to 2 nucleotides to be mismatched. **E**) QuiCAT shows the highest quality metrics compared to other extraction pipelines.
